# A novel virtual hospital at home model during the coronavirus disease 2019 (COVID-19) pandemic

**DOI:** 10.1017/ice.2020.435

**Published:** 2020-08-24

**Authors:** Patrick P. Ryan, Kellie L. Hawkins, Stacey Altman, Lisa Granatowski, Bradley D. Shy, Jeremy Long, Rebecca Hanratty

**Affiliations:** 1 Department of General Internal Medicine, Denver Health & Hospital Authority, Denver, Colorado; 2 University of Colorado School of Medicine, Aurora, Colorado; 3 Division of Infectious Disease, Department of Medicine, Denver Health & Hospital Authority, Denver, Colorado; 4 Ambulatory Care Services, Community Health Services, Denver Health & Hospital Authority, Denver, Colorado; 5 Department of Emergency Medicine, Denver Health & Hospital Authority, Denver, Colorado

The COVID-19 pandemic has prompted healthcare systems to rapidly adapt healthcare delivery to accommodate a novel infectious disease while considering infection control practices, hospital capacity, and continued management of other medical conditions. Additionally, the COVID-19 pandemic has disproportionately affected minority communities and those suffering from lower socioeconomic status in the United States; populations that already face worse outcomes in other chronic medical conditions such as hypertension, coronary artery disease, and diabetes.^1–3^

To prepare for possible surge capacity, we built a virtual hospital at home (VHH) to manage patients if the hospital could no longer accommodate new admissions. However, in response to early experiences with patients discharged from the emergency department (ED) who later decompensated, we pivoted to provide care to high-risk patients who did not meet the admission criteria.

In this report, we describe the development and implementation of a VHH program at an urban safety-net healthcare system to provide remote monitoring for high risk patients with COVID-19 and the early outcomes associated with this program.

**Fig. 1.**
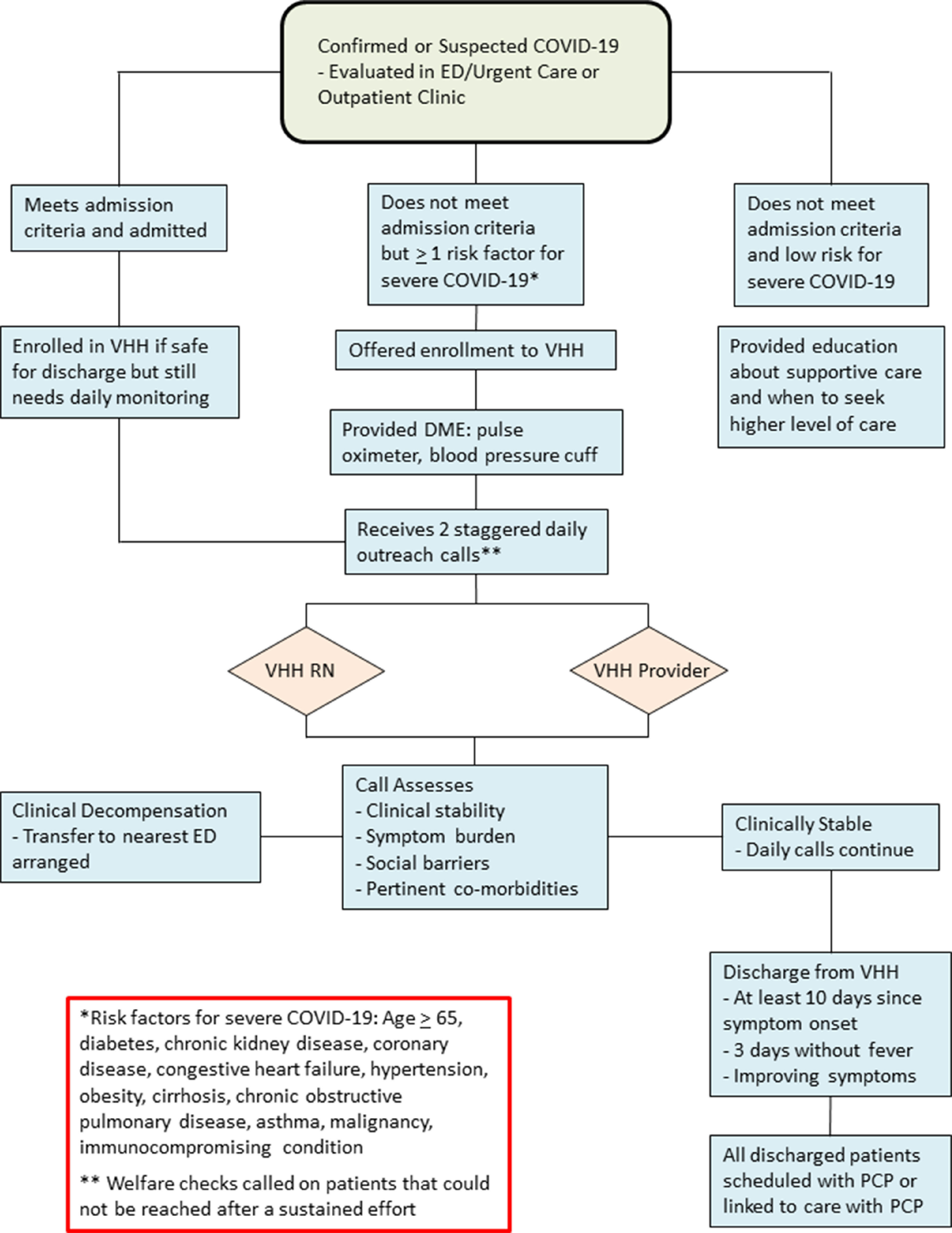
Flow Diagram of Virtual Hospital at Home Design and Protocol.

## Methods

### Objectives

The VHH was implemented to provide a safe home monitoring program for COVID-19 patients that did not meet admission criteria to provide an overflow contingency plan if our hospital reached capacity. Secondary goals included analyzing patient outcomes, and assessing the feasibility and acceptability of the VHH in a safety-net system.

### Setting and patient population

Denver Health is an integrated, safety-net healthcare system. Most patients have government insurance (54% Medicaid) or are uninsured (18%). More than half of patients are members of racial/ethnic minority groups: 37% are Hispanic and 14% are black.

### Virtual hospital at home design

Patients eligible for VHH monitoring include suspected or confirmed COVID-19 cases and at least 1 risk factor for disease complications but do not meet admission criteria (Fig. 1).^1^ VHH patients are provided a pulse oximeter and an automated blood pressure cuff to complete home monitoring assessments. VHH patients receive 2 phone calls per day: one from an RN and another from a provider to assess for interval symptom progression. VHH providers represent outpatient attending physicians, resident physicians, and advanced practice providers (ie, nurse practitioners and physician assistants).

Patients demonstrating clinical deterioration are transferred to the nearest ED via a coordinated effort between the VHH program and local EMS. Patients remaining clinically stable and meeting discharge criteria from the VHH are scheduled with their primary care provider (PCP) or receive assistance to establish care if they do not have a PCP.

This project was approved by the institutional quality improvement review committee.

## Results

In total, 233 patients were referred to VHH for home monitoring between April 3 and May 24, 2020. The mean patient age was 49 years, and the most common risk factors for decompensation were hypertension, obesity, and diabetes (Table 1).

**Table 1.** Patient Demographics and Outcomes

Characteristic	Overall No. (%)
**Age, mean y (SD)**	**49.2 (14.7)**
≥65	**34 (14.6)**
**Male**	108 (46.4)
**Does not have PCP**	53 (22.8)
**COVID-19 Status**	
Confirmed	129 (55.4)
Negative	72 (30.9)
Test Unavailable	32 (13.7)
**Race/Ethnicity**	
White	55 (23.6)
Black	22 (9.4)
Hispanic	145 (62.2)
Other	11 (4.7)
**Comorbid conditions**	
Diabetes mellitis	60 (25.8)
Coronary artery disease	9 (3.9)
Chronic heart failure	7 (3)
End-stage renal disease	2 (0.9)
Hyoertrension	142 (60.9)
Cirrhosis	4 (1.7)
Asthma/COPD	54 (23.2)
BMI >30	96 (41.2)
Malignancy	6 (2.6)
Immunosuppressed	23 (9.9)
**Referral source**	
Emergency department	80 (34.3)
Urgent care	57 (24.5)
Ambulatory testing	54 (23.2)
Hospitalist service	36 (15.5)
Other	6 (2.6)
**Preferred patient language**	
English	131 (56.2)
Spanish	94 (40.3)
Non-English/Non-Spanish	8 (3.4)
**VHH outcomes**	
Mean Duration of VHH Monitoring, d (SD)	4.1 (3.1)
Discharged from VHH	190 (81.6)
ED visit	19 (8.2)
Hospital admission	9 (3.9)
ICU admission	2 (0.9)
Unable to contact	13 (5.6)
**Insurance coverage**	
Commercial	37 (15.9)
Medicaid	89 (38.2)
Medicare	34 (14.6)
Uninsured	66 (28.3)
Unknown/Other	7 (3)

Note. SD, standard deviation; PCP, primary care provider; COPD, chronic obstructive pulmonary disease; BMI, body mass index; VHH, virtual hospital at home; ICU, intensive care unit.

The average duration of VHH monitoring was 4 days. Of enrolled patients, 190 were successfully discharged from the program, 31 (13.3%) required an escalated level of care, and 11 (35.4%) required admission (Table 1). The VHH was unable to contact 13 patients (5.6%). Most patients were uninsured (28.3%) or were covered by Colorado Medicaid (38.2%).

## Discussion

The VHH provided a safe and effective mechanism to remotely monitor a population that has been disproportionately affected by the COVID-19 pandemic. As 95% of patients referred participated, it seemed to be well received by patients, and the program successfully managed most patients within their own homes. Additionally, home-monitoring equipment was well received by patients referred to the VHH, and these tools helped care providers assess the need for care escalations.

The VHH also assisted in managing hospital capacities through 2 distinct routes. First, the VHH allowed ED providers a mechanism to refer patients to the home-monitoring program who may have otherwise been admitted to an observation inpatient stay, avoiding an influx of admissions for lower acuity patients. Second, the VHH provides hospitalists an outlet to safely discharge COVID-19 patients earlier, knowing that patients will have close outpatient oversight and guidance in their care.

Although prior hospital-at-home programs have used home visits as the care delivery mechanism, the VHH built upon these constructs exclusively through telehealth, while demonstrating program feasibility and safe patient care.^4,5^ By exclusively utilizing a telehealth approach, the VHH minimized the impact on inpatient PPE supplies during the COVID-19 pandemic.

Furthermore, this project demonstrates the acceptance of a novel telehealth program among a diverse patient population, most of whom were ethnic minorities and were either uninsured or relied on some form of government insurance. As the COVID-19 pandemic has disproportionately affected ethnic minority groups, more widespread implementation of telehealth home monitoring programs may even out this health disparity, but further study is needed to address this particular question.

Lastly, the VHH provides an opportunity to connect patients to longitudinal primary care. The VHH assisted unestablished patients to connect with enrollment services to implement medical coverage options, and it helped give patients direct access to scheduling in primary care clinics.

This evaluation has limitations. The program was designed to be a clinical intervention, not a prospective study, and our study cohort did not have a comparison group. Only patients with a functioning phone number could participate. The results are observational and may not be generalizable to other healthcare settings. Further study is necessary to understand differences in outcomes and the financial impact of VHH monitoring compared to observation hospitalizations for COVID-19 patients with similar risk profiles.
